# Use of artificial intelligence in activating the role of Saudi universities in joint scientific research between university teachers and students

**DOI:** 10.1371/journal.pone.0267301

**Published:** 2022-05-04

**Authors:** Aida Albasalah, Samar Alshawwa, Razan Alarnous

**Affiliations:** 1 Arabic Language Department, Arts College, Princes Nourah bint Abdulrahman University, Riyadh, Saudi Arabia; 2 Department of Pharmaceutical Sciences, College of Pharmacy, Princess Nourah bint Abdulrahman University, Riyadh, Saudi Arabia; 3 Child Development Center, King Abdullah bin Abdulaziz University Hospital, Princess Nourah bint Abdulrahman University, Riyadh, Saudi Arabia; Dai Hoc Duy Tan, VIET NAM

## Abstract

Scientific research in Saudi Arabia’s universities has undergone significant changes in recent years with the speed of higher education expansion and the opening of new universities. Artificial intelligence (AI) can be applied to existing data analysis processes to enhance pattern recognition and to support advanced data analysis. This study aimed to investigate the obstacles to activating the role of university instructors and students in joint scientific research. The study also aimed to evaluate joint scientific research between university teachers and students in universities, as well as the mechanisms for activating joint scientific research among male and female students in health and humanities science within Saudi universities, to enhance creation and invention achievements. To determine the obstacles to activating scientific research roles between students and tutors in Saudi universities using AI, a simple random sampling technique was adopted for this study. A well-structured questionnaire was administered to 250 respondents affiliated with universities in Saudi Arabia. The data collected were statistically analyzed with the aid of the Statistical Package for Social Science (SPSS) version 20. The results of this study revealed that the objectives of joint scientific research between university teachers and students in universities have a significant positive predictor of obstacles to activating the role of teachers in joint scientific research with students in Saudi universities. The study also showed that there was a statistically significant correlation (*p value* = 0.00) between each of the variables.

## Introduction

Artificial intelligence (AI) can be defined as the property of machines that imitates human intelligence, which is characterized by behaviors such as cognitive abilities, memory, learning, and decision-making [1]. AI also refers to the artificial creation of human intelligence capable of learning, perceiving, and processing information. It is rapidly becoming a prevailing instrument to solve the problem of image recognition and document classification, as well as for the advancement of interdisciplinary issues [2].

AI is considered the strategic driver of the future (by the Chinese government and the governments of other countries) and provides strategic development plans and promotes inclusive progress at the national level to organize for the coming AI society [3].

According to the Katz School of Health and Science [4], “Artificial intelligence promises to provide some of the most important and disruptive innovations of this century. Self-driving cars, robotic assistants, and automated disease diagnostics are all products of an emerging AI revolution that will restructure the way we live and work. And with the request for talented engineers more than doubling in recent years, there are countless opportunities for professionals looking to work in AI research and development”.

AI has emerged as the center of attention in all sectors of society. It has existed for sixty-five years, as the idea of AI was once first suggested in 1956. Since then, the development of AI has experienced ups and downs. It has gradually begun to heat up and has become a common search target for governments, industries, research institutions, and the consumer market. With the development of AI strategies and capital markets in several countries, AI companies or enterprises, products, and services continue to emerge [3].

In recent years, tech giants have continuously created AI research laboratories, invested more resources to conquer the AI market, and even completely transformed into AI. Companies rely on AI to plan in depth for their futures. AI is naturally situated between sciences and human sciences, which requires not only the contribution of statistics, mathematics, mathematical logic, neurosciences, and computer science, but also that of health sciences, philosophy, psychology, cognitive sciences, sociology, law, and so on.

Whether engaging in the renaissance and development of interdisciplinary scientific research across law, ethics, health, public policy, and other humanities, or the research and development of AI technology, all must hold AI in awe. The interdisciplinary quality of AI in education (AIED) exhibits challenges for researchers but can be implemented in various teaching settings [5].

This study aimed to investigate the obstacles to activating the role of university teachers in joint scientific research in the health and humanities sciences. The study also aimed to evaluate joint scientific research in the health and humanities sciences between tutors and students in universities, the mechanisms for activating joint scientific research among male and female students, and the obstacles to activating the scientific research role between students and university teachers in the in health and humanities sciences in Saudi universities by using AI.

### Research questions

- What are the obstacles to activating the role of university teachers in joint scientific research in the health and humanities sciences in Saudi universities?
- What are the objectives of joint scientific research between university teachers and students in the health and humanities sciences?
- What are the mechanisms for activating joint scientific research among male and female students in universities in the health and humanities sciences?
- What are the obstacles to activating the scientific research role between students and tutors in health and humanities sciences in Saudi universities?

#### Definition of terms

*Artificial intelligence*. A way of making a computer, computer-controlled robot, or software think intelligently in the same way humans think.

*University*. An organization of higher learning that provides facilities for teaching and research.

*Scientific research*. Research conducted in a planned manner with the aim of contributing to science through the systematic collection, interpretation, and evaluation of data.

*Science*. A branch of knowledge that deals with a body of facts.

*Research*. Diligent and systematic analysis or investigation into a subject to discover facts, theories, etc.

## Literature review

The term “artificial intelligence” stems from the 1950s. According to the father of AI, John McCarthy (an American computer scientist), and his colleagues, it was proposed at a Dartmouth conference in 1956 that “artificial intelligence is a way of making a computer, computer-controlled robot, or software think intelligently, in the same way intelligent humans think” [6, 7].

Over the next 65 years, AI has had various ups and downs. In addition to the unceasing development of the direction of the technology itself, AI is also acquiring different levels of significance for flexibility in interpretation. Before “AlphaGo” beat “Lee Sedol” and “Ke Jie,” AI, the exposure of most people was limited to what they saw in movies. Over the decades, a series of films, such as Artificial Intelligence, The Matrix, Her, and The Incredibles, have explained human aspiration and fear of AI. The idea of AI is not only scientific common sense, but also a method of popular commercial culture [3].

Scientific research in Saudi Arabia’s universities has undergone significant changes in recent years, with the increased speed of higher education expansion and the opening of new universities. This includes promoting the scientific research movement and innovation, as one of the main functions of universities, and doubling the interest of universities and scientific research, patents, and diverse sources of income and expenditure for scientific research and development in Saudi universities through the generous support of King Abdulaziz City for Science and Technology (KAUST) through the National Science, Technology, and Innovation Plan (NSTIP), as well as private sector support representing privileged science chair, “SABIC chair” in Arab universities. Saudi Arabia is the best example, and a worldwide network of research centers and offices are managed by ARAMCO, representing areas of expertise, innovation capabilities, and new and strategic research in desired fields around the world [8].

### What do people think of AI when it is mentioned?

Product designers, entrepreneurs, policy makers, and the general public commonly use the term “AI” in a variety of contexts. Some people refer to AI as AlphaGo and robots. “Autonomous vehicles,” “terminators,” “series,” “big data,” etc., are also common words. When talking about AI, people often confuse it with the concept of robots. However, like “cloud computing,” “big data,” and “machine learning,” the term AI has been used unrestrictedly by marketers and advertising copywriters. In the eyes of other groups, AI is a panacea and looks like a time bomb that causes massive unemployment.

### AI and interdisciplinary scientific research

AI can be applied to existing data analysis processes to boost pattern recognition and support advanced data analysis. As examples of this already exist in various fields of research, and more access is given to advanced data techniques and computational powers, AI can become a valuable tool for any researcher. This may require changing the technical makeup of research teams or creating new forms of collaboration between teams and between academia and industry. It provides access to both the advanced data science skills required for the application of AI and the computational power to build AI systems [2].

The relationship between AI and interdisciplinary scientific research is considered a two-way road. Although the direction in which it was established is better known (applying AI to other areas or fields), both directions are considered here, from AI to other areas or fields, and from other areas to AI.

Applying knowledge from other areas to AI development is essential to move forward and achieve the full potential of the AI revolution.

### From AI to other areas or fields

Quantitative science, medical care, biology, economics, and finance, for example, using AI to make predictions or decisions, have been widespread and perhaps experienced overkill in the last few years. Applying AI to these areas is still an active area of research, but we believe that the biggest challenge for the future of AI is ahead. AI solutions need to be developed to perform exploratory analysis rather than simply guessing or making decisions so as to find new, attention-grabbing patterns in complex systems or facilitate scientific discovery [9]. Chen et al.’s study indicated the importance of AIED research and highlighted the application of AI with educational theories. They also highlighted the implementation of launching a new Elsevier journal related to AIED [10, 11].

One standard methodology in the field of neuroscience is understanding what AI model best predicts behavioral data, for example, humans to support or inform hypotheses of the structure and function of biological cognitive systems [12].

In this case, the process of training the AI agent itself is an experiment, as the essential concern is not the performance of the basic algorithm, but the ability to explain cognitive function. Is it possible to create an AI algorithm that exchanges all stages of the scientific process, from asking questions to creating data to analyzing and interpreting results? These auto-detections are considered by some experts to be the ultimate goal, but are currently unattainable [13].

### From other fields to AI

Although AI methods can easily affect many areas of science, they continue to benefit from insights from areas such as neuroscience [14–17], seen in the similarity between machine and human facial recognition [18] and the use of the concept of facial space in deep convolutional neural networks [17, 19]. Other areas that influence AI research include evolutionary biology [20] and quantum mechanics [21]. According to Rosenblatt (1958), as stated in a study carried out by Kusters et al. [22], “One of the biggest successes of integrating insights from other fields in modern day AI, the perceptron, became the prelude to the modern neural networks of today.” Perceptrons and neural networks can be considered highly simplified cortical neural circuit models.

AI systems are still far from being compared with human intelligence, and several questions remain unanswered. For example, how can an AI system learn and generalize while being visible to only a small amount of data? How can we bridge the gap between low-level neural mechanics and high-level symbolic reasoning?

Although AI algorithms still focus primarily on the modeling of pure cognitive processes (e.g., learning, abstraction, planning, etc.), complementary approaches bring intelligence as an emerging attribute of the cognitive system, including morphology, sensorimotor, development, and social, cultural, and evolutionary processes. This method is inspired by numerous scientific fields, such as evolutionary biology, developmental science, and behavioral ecology. The latest advances in reinforcement learning have taken some steps in this direction. Agents who can autonomously decompose complex tasks into simpler tasks (automatic courses) can develop more complex behaviors through collaborative adaptation in an environment of hybrid cooperative competition [23].

Finally, recent studies have proposed the joint production of increasingly complex and diverse learning environments and results as a way to realize open learning [24]. One of the relevant research directions is the study of continuous learning systems in lifelong learning [25]. Other research has focused on the application of AI in language education domains, including reading, writing, and vocabulary acquisition [26]. Hwang and Tue also reported advancements in AI in mathematics research [27].

That is, they learn continuously without facing the well-known phenomenon of forgetting [28]. Combining these studies raises the following questions: How can we take advantage of recent advances in deploying AI agents in realistic ecosystems? How will the dynamics of these systems drive the acquisition of progressively complex skills?

### Role of Saudi universities in scientific research

Saudi Arabia has given importance to national development plans to achieve significant progress in the study of AI. This process must be measured by the number of research papers published in international scientific journals, as well as approved patents. Universities have rich international scientific research functions. The quantity and quality of research products are evaluated by Saudi Universities and King Abdul Aziz City in Science and Technology, as well as in some government agencies or organizations and semi-distribution products that support Saudi studies, problems, and interests for questions, especially in connection with the implementation of the Saudi Vision 2030.

## Research method

To facilitate the collection of data for this study, a structured questionnaire was administered to individuals associated with universities in Saudi Arabia. Two hundred and fifty appropriately filled out questionnaires were retrieved. The respondents in the study were university students at the master’s and doctoral levels, members of teaching staff and administrative board members of universities in Saudi Arabia.

A simple random sampling technique was utilized for this study. The survey design included questions related to the respondent’s sociodemographic characteristics, obstacles (first axis) and objectives (second axis) to activating the role of university teachers in joint scientific research in health and humanity sciences in Saudi universities, mechanisms for activating joint scientific research among male and female students in universities in health and humanities sciences (third axis), and the obstacles to activating the joint scientific research role between the student and the university teacher in health and humanities sciences in Saudi universities (fourth axis). All of research aims would be implemented by using AI.

The data collected were statistically analyzed using the Statistical Packages for Social Science (SPSS) version 20. Descriptive statistics were used to report the frequencies and percentages of definite variables. The data were also subjected to regression (ordinal regression) and correlation (Spearman’s rank) analysis to determine the relationship between the four axes.

## Results

Table 1 above shows the sociodemographic characteristics of the respondents in this study. The table contains the gender, age, position, job title, specialization, years of experience, and the regions of the respondents.

**Table 1:** Socio-demographic characteristics of the respondents.

VARIABLES	FREQUENCY	PERCENTAGE
**GENDER**		
Male	40	16.0
Female	210	84.0
**Total**	**250**	**100.0**
**AGE**		
18–24 years	27	10.8
25–34 years	40	16.0
35–44 years	87	34.8
45–54 years	64	25.6
55 years and above	32	12.8
**Total**	**250**	**100.0**
**POSITION**		
Professor	53	21.2
Assistant Professor	66	26.4
Associate-Professor	35	14.0
Lecturer	64	25.6
Teaching Assistant	32	12.8
**Total**	**250**	**100.0**
**JOB TITLE**		
Student/University Student	54	21.6
Teaching Staff Member	148	59.2
Administrative Board Member	37	14.8
Master/PhD Student	11	4.4
**Total**	**250**	**100.0**
**SPECIALIZATION**		
Faculty of Humanities	148	59.2
Faculty of Science	59	23.6
Community College	25	10.0
College of Health Science	18	7.2
**Total**	**250**	**100.0**
**Years of Experience**		
Less than 5 years	74	29.6
5–10 years	57	22.8
More than 10 years	119	47.6
**Total**	**250**	**100.0**
**Region**		
Central Region	199	79.6
Western Region	14	5.6
Eastern Region	28	11.2
Southern Region	5	2.0
Northern Region	4	1.6
**Total**	**250**	**100.0**

The majority (210 or 84.0%) of the respondents were female, while 40 (16.0%) of the respondents were male. Twenty-seven (10.8%) of the respondents were between the ages of 18–24 years, 40 (16.0%) were between the ages of 25–34 years, 87 (34.8%) of the respondents were between the ages of 35–44 years, 64 (25.6%) of the respondents were between the ages of 45–54 years, and 32 (12.8%) of the respondents were aged 55 years and above.

Fifty-three (21.2%) of the respondents were professors, 66 (26.4%) were assistant professors, 35 (14.0%) were associate professors, 64 (25.6%) were lecturers, and 32 (12.8%) were teaching assistants at their respective universities.

Most of the respondents 148 (59.2%) were teaching staff members, 54 (21.6%) were students/university students, 37 (14.8%) were administrative board members, and 11 (4.4%) were master/PhD students.

Also represented in the table above are the regions of the respondents. The majority (199 or 79.6%) were from the *Central Region* of Saudi Arabia, while the rest were scattered across other regions: the *Western Region* (14 or 5.6%), the *Eastern Region* (28 or 11.2%), the *Southern Region* (5 or 2.0%), and the *Northern Region* (4 or 1.6%).

**Fig 1:**
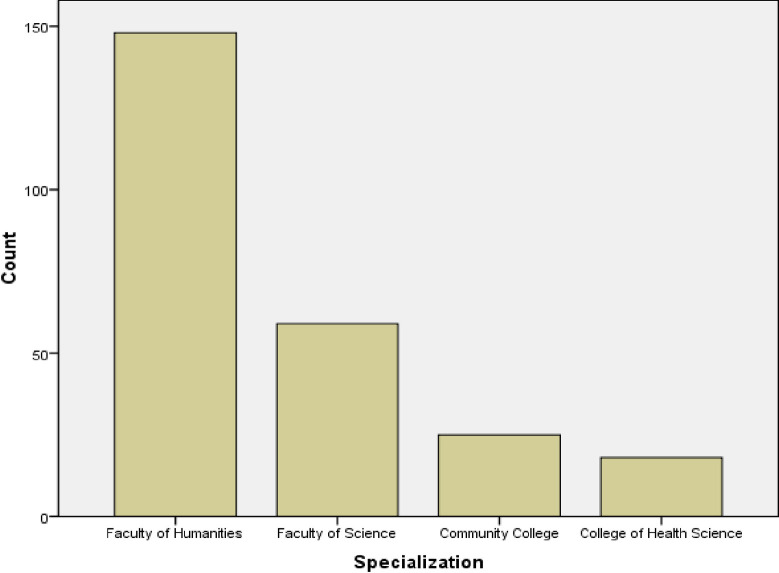
Sociodemographical features showing specialization of respondents.

Fig 1 below shows that the majority (148 or 59.2%) of the respondents were from the faculty of humanities, 59 (23.6%) were from the faculty of science, 25 (10.0%) were from a community college, and 18 (7.2%) of the respondents were from the college of health science.

**Fig 2:**
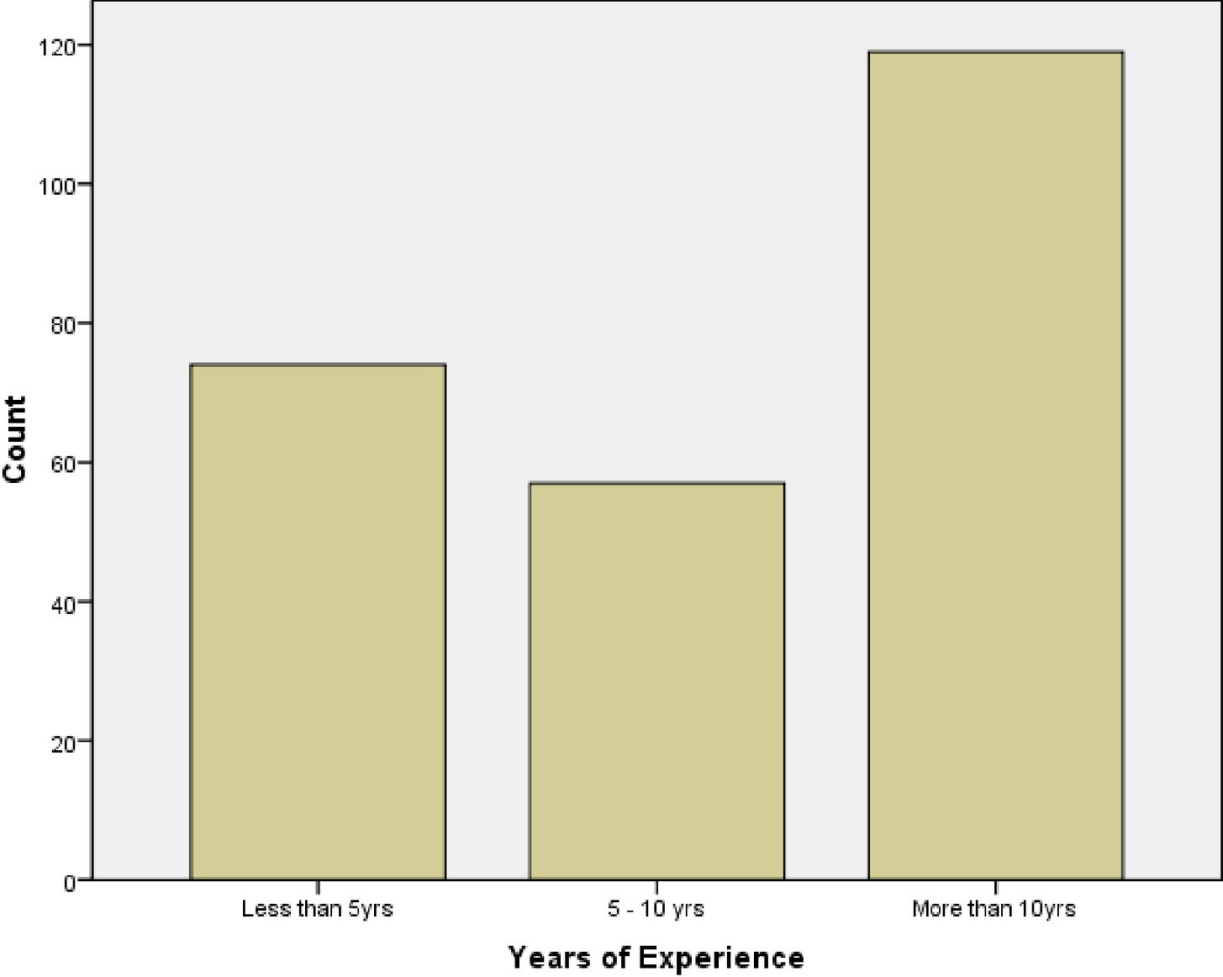
Sociodemographic features showing years of experience of respondents.

From Fig 2 below, 119 (47.6%) of the respondents had more than ten years of experience, 74 (29.6%) had less than five years of experience, and 57 (22.8%) had five to ten years of experience.

From Table 2 above, the objectives of the joint scientific research between university teachers and students in health and humanities sciences is a significant positive predictor of obstacles to activating the role of university teachers in joint scientific research. Thus, for every one-unit increase in the objectives, there is a predicted increase of 1.535 in the long odds of being at a higher level in obstacles to activating the role of university teacher.

**Table 2:** Parameter estimate.

Parameter Estimates								
		Estimate	Std. Error	Wald	df	Sig.	95% Confidence Interval	
							Lower Bound	Upper Bound
Threshold	[FA = 2.12]	4.789	1.283	13.926	1	.000	2.274	7.304
	[FA = 2.24]	5.531	1.127	24.082	1	.000	3.322	7.740
	[FA = 2.29]	6.005	1.070	31.492	1	.000	3.908	8.102
	[FA = 2.41]	6.349	1.043	37.068	1	.000	4.305	8.392
	[FA = 2.47]	6.612	1.028	41.389	1	.000	4.598	8.627
	[FA = 2.65]	7.004	1.013	47.801	1	.000	5.019	8.990
	[FA = 2.76]	7.301	1.007	52.614	1	.000	5.328	9.274
	[FA = 2.82]	7.431	1.005	54.698	1	.000	5.462	9.400
	[FA = 2.88]	7.760	1.003	59.907	1	.000	5.795	9.726
	[FA = 2.94]	7.856	1.003	61.402	1	.000	5.891	9.821
	[FA = 3.00]	8.256	1.005	67.511	1	.000	6.287	10.226
	[FA = 3.06]	8.649	1.010	73.291	1	.000	6.669	10.629
	[FA = 3.12]	8.709	1.011	74.163	1	.000	6.727	10.691
	[FA = 3.18]	9.038	1.018	78.821	1	.000	7.043	11.033
	[FA = 3.24]	9.189	1.022	80.910	1	.000	7.187	11.191
	[FA = 3.29]	9.419	1.027	84.057	1	.000	7.406	11.433
	[FA = 3.35]	9.588	1.032	86.315	1	.000	7.565	11.610
	[FA = 3.41]	9.778	1.037	88.840	1	.000	7.745	11.811
	[FA = 3.47]	9.881	1.040	90.197	1	.000	7.842	11.920
	[FA = 3.53]	10.132	1.048	93.465	1	.000	8.078	12.186
	[FA = 3.59]	10.420	1.057	97.182	1	.000	8.348	12.492
	[FA = 3.65]	10.771	1.068	101.658	1	.000	8.677	12.865
	[FA = 3.71]	10.954	1.074	103.972	1	.000	8.848	13.059
	[FA = 3.76]	11.231	1.084	107.445	1	.000	9.107	13.355
	[FA = 3.82]	11.424	1.090	109.837	1	.000	9.288	13.560
	[FA = 3.88]	11.616	1.097	112.200	1	.000	9.467	13.766
	[FA = 3.94]	11.808	1.103	114.531	1	.000	9.646	13.971
	[FA = 4.00]	12.125	1.115	118.308	1	.000	9.940	14.309
	[FA = 4.06]	12.274	1.120	120.070	1	.000	10.078	14.469
	[FA = 4.12]	12.574	1.131	123.582	1	.000	10.357	14.791
	[FA = 4.18]	12.728	1.137	125.370	1	.000	10.500	14.956
	[FA = 4.24]	12.941	1.145	127.851	1	.000	10.698	15.185
	[FA = 4.29]	13.192	1.154	130.778	1	.000	10.931	15.453
	[FA = 4.35]	13.466	1.163	134.004	1	.000	11.186	15.746
	[FA = 4.41]	13.779	1.174	137.774	1	.000	11.478	16.080
	[FA = 4.47]	14.043	1.182	141.040	1	.000	11.726	16.361
	[FA = 4.53]	14.454	1.195	146.266	1	.000	12.111	16.796
	[FA = 4.59]	14.590	1.199	148.047	1	.000	12.240	16.940
	[FA = 4.65]	14.734	1.203	149.938	1	.000	12.376	17.093
	[FA = 4.71]	14.887	1.208	151.953	1	.000	12.520	17.254
	[FA = 4.76]	15.300	1.220	157.321	1	.000	12.909	17.691
	[FA = 4.82]	15.660	1.231	161.718	1	.000	13.246	18.073
	[FA = 4.88]	16.018	1.245	165.512	1	.000	13.577	18.458
	[FA = 4.94]	16.519	1.271	169.046	1	.000	14.029	19.009
Location	SA	1.535	.337	20.795	1	.000	.875	2.195
	TA	-.933	.359	6.768	1	.009	-1.636	-.230
	FTA	2.292	.233	97.013	1	.000	1.836	2.749

Link function: Logit.

**FA = First Axis:** Obstacles to activating the role of university teachers in joint scientific research in health and humanities sciences in Saudi universities

**SA = Second Axis:** The objectives of the joint scientific research between the university teacher and the student in health and humanities sciences at the universities

**TA = Third Axis**: Mechanisms for activating joint scientific research among male and female students in universities in health and humanities sciences

**FTA = Fourth Axis:** The obstacles to activating the joint scientific research role between students and university teachers in the health and humanities sciences in Saudi universities.

Mechanisms for activating joint scientific research among male and female students in universities is a negative significant predictor of the obstacles to activating the role of university teacher in joint scientific research in health and humanities sciences in Saudi universities. The negative coefficient value of -0.933 shows that for every one-unit increase in mechanisms, there is a predicted decrease of 0.993 in the long odds of being at a higher level on the obstacles to activating the role of university teacher.

The obstacles to activating the joint scientific research role between students and university teachers in health and humanities sciences in Saudi universities is a significant positive predictor (2.292) of the obstacles to activating the role of university teacher. Thus, for every one-unit increase in the obstacles to activating the joint scientific research role, there is a predicted increase of 2.292 in the long odds of being at a higher level in the obstacles to activating the role of university teacher.

From Table 3 above, the correlation between the first and second axis is 0.530, which indicates a moderate relationship between the two. There is a p value of 0.000, which is less than 0.05, and it indicates that there is a statistically significant correlation between the dependent variable (obstacles) and the independent variable (objectives).

**Table 3:** Correlation among the four variables.

Correlations						
			FA	SA	TA	FTA
Spearman’s rho	FA	Correlation Coefficient	1.000	.530**	.435**	.676**
		Sig. (2-tailed)	.	.000	.000	.000
		N	250	250	250	250
	SA	Correlation Coefficient	.530**	1.000	.822**	.567**
		Sig. (2-tailed)	.000	.	.000	.000
		N	250	250	250	250
	TA	Correlation Coefficient	.435**	.822**	1.000	.590**
		Sig. (2-tailed)	.000	.000	.	.000
		N	250	250	250	250
	FTA	Correlation Coefficient	.676**	.567**	.590**	1.000
		Sig. (2-tailed)	.000	.000	.000	.
		N	250	250	250	250

**. Correlation is significant at the 0.01 level (2-tailed).

**FA = First Axis:** Obstacles to activating the role of university teachers in joint scientific research in health and humanities sciences in Saudi universities

**SA = Second Axis:** The objectives of the joint scientific research between the university teacher and the student in health and humanities sciences in the universities

**TA = Third Axis**: Mechanisms for activating joint scientific research among male and female students in universities in health and humanities sciences

**FTA = Fourth Axis:** The obstacles to activating the joint scientific research role between the student and the university teachers in health and humanities sciences in Saudi universities

The Spearman correlation value of the first and third axis is 0.435, also indicating a moderate correlation between them. The p value of 0.000 is less than 0.05, and it indicates that there is a significant correlation between the first axis (dependent variable) and the third axis (independent variable).

There is a strong correlation (r = 0.676) between the obstacles to activating the role of university teachers in joint scientific research in health and humanities sciences in the Saudi universities and the obstacles to activating the joint scientific research role between the student and the university teacher. There is a statistically significant correlation between the first axis and the fourth axis, since the p value of 0.000 is less than 0.005.

There is a very strong correlation (r = 0.822) between the objectives of joint scientific research between the university teacher and the student and the mechanisms for activating joint scientific research among male and female students. The p value of 0.000 is less than 0.05, which indicates that the correlation coefficient between the second and third axes is significant.

The correlation between the second and fourth axes is 0.567, which indicates that there is a moderate relationship between them. There is a statistically significant correlation between the second axis (the objectives of the joint scientific research between university teachers and the students in universities) and the fourth axis (the obstacles to activating the joint scientific research role between the students and university teachers in Saudi universities), since the p value (0.000) is less than 0.05.

There is a strong correlation (r = 0.590) between mechanisms for activating joint scientific research among male and female students in health and humanities sciences in universities and the obstacles to activating the joint scientific research role between students and university teachers. There is a statistically significant correlation between the third axis (mechanisms for activating joint scientific research among male and female students) and the fourth axis (the obstacles to activating the joint scientific research role), as the p value of 0.000 is less than 0.05.

## Discussion

AI helps to learn, perceive, and process information, which is an instrument for solving image recognition and document classification, and for the advancement of interdisciplinary problems [29] which is in line with the objectives of joint scientific research in health and humanities sciences. Furthermore, the obstacles or challenges to activating the role of university teachers in joint scientific research may be a result of barriers in communication, perhaps due to differences in culture, technology, and interest [22]. Other obstacles may include lack of qualified faculty members to prepare joint research using AI, lack of educational means and modern educational technology, ignoring the real role of university education, which is to promote scientific research with AI to solve the nation’s problems, etc. Hwang et al. implemented an adaptive learning model that improves students’ learning achievements and minimizes their mathematical challenges [30].

In line with the mechanisms for activating joint scientific research among male and female students in universities in health and humanities sciences, Akulenko [29] properly organized and structured the scientific research of students in one of his articles, “The effect of university student scientific research in training future professionals,” into the following functions: instructional, structuring-based, analytical and correction, conative, stimulating, and educational.

In line with the obstacles to activating the scientific research role between students and university teachers, Babamohamadi et al. [31] said, “The approach and function of university students to research process: A cross sectional study,” noting that the problem is that students are not eager to engage in practicing research methods, learning more about innovative techniques of solving scientific issues, and knowing how to analyze various information flows because they fair difficult challenges [32].

## Conclusion

AI is always present in science and society. It inevitably interacts with other fields of science, and in this study, we examined the obstacles to activating the role of university teachers in joint scientific research and between university teachers and students in health and humanities sciences in universities, the mechanisms for activating joint scientific research among male and female students, and the obstacles to activating the scientific research role between students and university teachers in Saudi universities by using AI.

The study shows that the objectives of joint scientific research between university teachers and students is a significant positive predictor of the obstacles to activating the role of university teacher in joint scientific research. Thus, an increase in objectives will result in an increase in obstacles.

Also, the study shows that the mechanisms for activating joint scientific research among male and female students in health and humanities sciences in universities is a negative significant predictor of the obstacles to activating the role of the university teacher in joint scientific research.

The study further revealed that the obstacles to activating the joint scientific research role between students and university teachers in health and humanities sciences in Saudi universities is a significant positive predictor of the obstacles to activating the role of university teachers in joint scientific research.

Lastly, the study indicated a moderate relationship between obstacles to activating the role of university teacher in joint scientific research in health and humanities sciences in Saudi universities and the objectives of the joint scientific research between university teachers and students, as well as a moderate correlation between the obstacles to activating the role of university teacher in joint scientific research and the mechanisms for activating joint scientific research among male and female students. There is a strong correlation between the obstacles to activating the role of university teachers and the obstacles to activating the joint scientific research role between students and university teachers. There is a very strong correlation between the objectives of the joint scientific research between university teachers and the students in health and humanities sciences in the universities and the mechanisms for activating joint scientific research among male and female students. A moderate relationship exists between the objectives of the joint scientific research between university teachers and students and the obstacles to activating the joint scientific research role between them, as well as a strong correlation between mechanisms for activating joint scientific research among male and female students and the obstacles to activating the joint scientific research role between students and university teachers.
